# Paradoxical Evidence Integration in Rapid Decision Processes

**DOI:** 10.1371/journal.pcbi.1002382

**Published:** 2012-02-16

**Authors:** Johannes Rüter, Nicolas Marcille, Henning Sprekeler, Wulfram Gerstner, Michael H. Herzog

**Affiliations:** 1 Laboratory of Psychophysics, Brain Mind Institute, École Polytechnique Fédérale de Lausanne, Lausanne, Switzerland; 2 Laboratory of Computational Neuroscience, Brain Mind Institute and School of Computer and Communication Sciences, École Polytechnique Fédérale de Lausanne, Lausanne, Switzerland; Indiana University, United States of America

## Abstract

Decisions about noisy stimuli require evidence integration over time. Traditionally, evidence integration and decision making are described as a one-stage process: a decision is made when evidence for the presence of a stimulus crosses a threshold. Here, we show that one-stage models cannot explain psychophysical experiments on feature fusion, where two visual stimuli are presented in rapid succession. Paradoxically, the second stimulus biases decisions more strongly than the first one, contrary to predictions of one-stage models and intuition. We present a two-stage model where sensory information is integrated and buffered before it is fed into a drift diffusion process. The model is tested in a series of psychophysical experiments and explains both accuracy and reaction time distributions.

## Author Summary

In models of decision making, evidence is accumulated until it crosses a threshold. The amount of evidence is directly related to the strength of the sensory input for the decision alternatives. Such one-stage models predict that if two stimulus alternatives are presented in succession, the stimulus alternative presented first dominates the decision, as the accumulated evidence will reach the threshold for this alternative first. Here, we show that for short stimulus durations decision making is not dominated by the first, but by the second stimulus. This result cannot be explained by classical one-stage decision models. We present a two-stage model where sensory input is first integrated before its outcome is fed into a classical decision process.

## Introduction

Decision making is of crucial interest in many disciplines such as psychology [1], [2], neuroscience [3]–[5], economics [6], [7], and machine learning [8]. Binary decision theories relate to situations where an observer (or machine) is confronted with one of two possible noisy stimuli ‘A’ and ‘B’. A decision has to be made whether ‘A’ or ‘B’ is present. For example, human readers have to decide whether a handwritten character is an 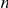 or a 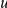; a trader has to decide whether to sell or to keep; a monkey has to decide whether dots on a screen are moving to the left or to the right [9]. While engineering and economical decision theories focus on how to compute optimal decisions [6], [7], [10], psychology and neuroscience investigate the actual decision making process in humans and animals [9], [11]–[14].

Decision making is usually assumed to be a one-stage process where evidence integration and decision making are identical (but see [15], [16]). In a standard accumulator model each bit of evidence is integrated and a decision is reached once the accumulated evidence for one of the two response alternatives crosses a threshold [13], [14], [17]–[33]. If the evidence itself is noisy, then the accumulation of evidence for each of the two stimulus alternatives leads to a diffusion-like process. For example, in the well-known random motion paradigm [9], moving dots appear at random moments in time, so that evidence for leftward or rightward moments arrives probabilistically and the accumulator is expected to evolve along a stochastic path that can be approximated by a drift-diffusion process. This is in good accordance with experimental studies where neurons in the macaque lateral intraparietal cortex (LIP) increase firing rates along a noisy trajectory up to the moment of decision [9], [32]–[34]. Since evidence is very noisy in this case, and arrives slowly over time, the decision process is rather slow [9]. Most experimental [9], [11], [12] and theoretical work on decision making [5]–[8] focuses on paradigms where noisy stimuli are presented for long durations, e.g. until a response is elicited (for exceptions see [31], [32]).

In other paradigms, where stimuli are less noisy, decisions can be extremely fast. For example, humans only need a fraction of a second to recognize objects such as animals in a picture [35]. This astonishing speed is also evident in sports such as table tennis or soccer requiring rapid reactions to moving balls. In these examples, the brain has to decide rapidly upon visual information available for only a hundred milliseconds or less. Note that even in these scenarios where stimuli are of high contrast (“low noise”), the responses of the observers can still be “noisy”.

Here, we first show psychophysically that one-stage models of the noisy accumulator or drift-diffusion type cannot explain the results of feature fusion experiments where two stimulus alternatives are presented in rapid succession for durations in the range of 20–160 ms. Second, we propose, instead, a two-stage model, where evidence integration is separated from a noisy drift-diffusion decision making process. Our results reveal additional aspects of the dynamics of decision making that are hidden in standard experimental paradigms where only one stimulus alternative is presented per trial.

## Results

**Figure 1:**
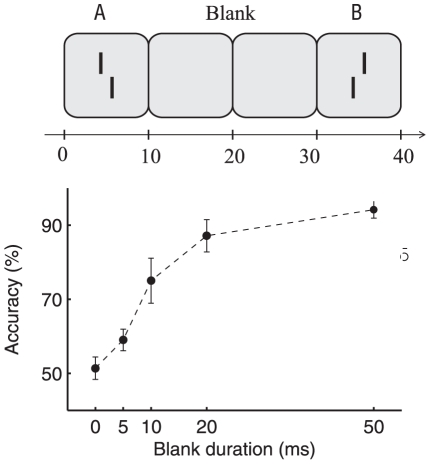
Reference Experiment. *Upper panel:* A left or right offset vernier was presented for 10 ms followed by a variable blank background (ISI, here shown for 20 ms) and, then, by a second vernier for 10 ms. *Lower panel:* Observers were asked to indicate whether the first or second vernier was offset to the right. Performance improves quickly with increasing ISI, reaching ceiling performance at 50 ms.

In our psychophysical experiments we worked with visual stimuli comprising two vertical bars with a small horizontal offset either to the left or to the right (Vernier stimulus, Figure 1). The contrast of the bars and the horizontal offset was chosen such that, after flashing the stimulus for 10 ms, human observers can reliably identify (accuracy above 90 percent correct) whether the lower vertical bar is offset to the left or right with respect to the upper vertical bar.

Next, we presented a sequence of two Vernier stimuli. As a reference we performed an experiment where the presentation of a first Vernier stimulus ‘A’ for 10 ms was separated from the presentation of a second Vernier stimulus ‘B’ by an interstimulus interval (ISI; blank screen) of variable duration. If the ISI was 50 ms, observers easily distinguished the two stimuli and could report, with an accuracy of above 90 percent correct, whether the first or the second Vernier stimulus was offset to the right. If the interstimulus interval was shorter, the accuracy dropped (Figure 1). The high precision of the subjects in spite of the short stimulus duration suggests that – in contrast to e.g. traditional random dot stimuli – the stimulus is highly informative with relatively little stimulus noise.

**Figure 2:**
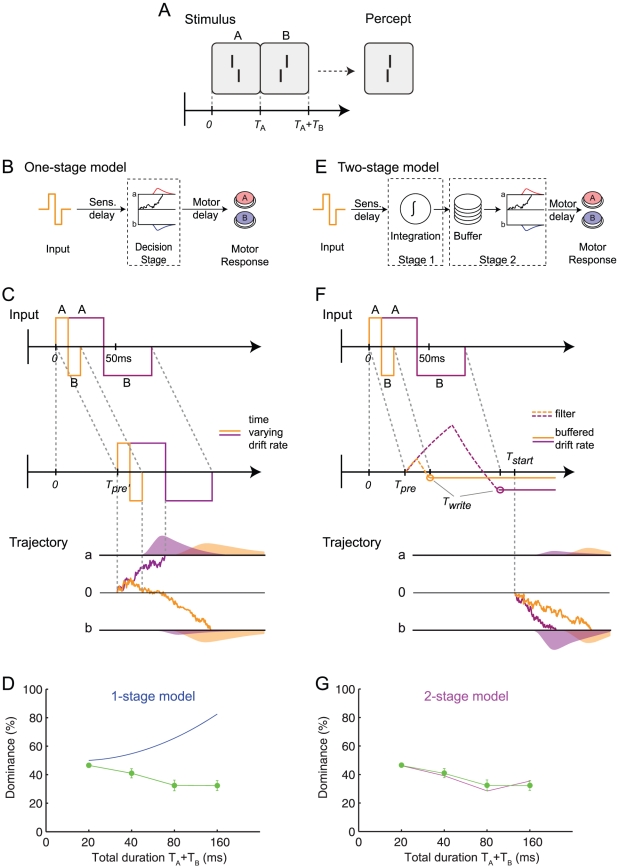
One-stage and two-stage models of decision making. (**A**) A vernier (stimulus ‘A’) is followed by a second vernier (stimulus ‘B’). The first vernier is either offet to the right (as shown) or to the left (not shown). The second vernier stimulus is always offset to the opposite side. Only one vernier is perceived and the offsets of the two vernier stimuli fuse. The perceived offset of the fused vernier is more strongly influenced by the second than the first vernier when the duration 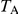 and 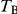 of stimulus ‘A’ and ‘B’ are equal, 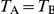. (**B**) One-stage model. After a sensory delay, the stimulus input is directly fed into the decision stage as the drift rate of a decision variable which is subject to a random walk. When the decision variable hits the upper boundary (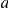), the decision is for the offset of the first vernier (stimulus ‘A’). When it hits the lower boundary (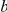), the decision is for the offset of the second vernier (stimulus ‘B’). A motor response is executed accordingly. Variability in the drift leads to different reaction times (red and blue curves show reaction time distributions). It is important to note that observers push one button for left responses and one for right responses. In this figure, however, button ‘A’ is a symbol denoting responses according to the first vernier stimulus (either left or right) and button ‘B’ according to the second vernier stimulus. (**C**) *Upper panel:* After preprocessing and signal transmission of duration 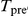 (sensory delay), the one-stage model translates the time course of the input directly into a time-varying drift rate of the decision process. *Bottom panel:* The time-varying drift rate directly drives the drift-diffusion process leading to trajectories which first increase and then decrease (orange trajectory, 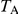 = 10 ms; purple, 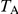 = 40 ms). The earlier the decision variable hits one of the boundaries, the faster the reaction times. For short 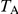 (e.g. 10 ms) the trajectory does not reach any of the boundaries (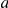, 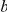) during stimulus presentation. One of the boundaries is reached after a random walk (orange line and reaction time distributions). For longer durations, the trajectory (purple line) more likely hits the upper than the lower boundary, leading to a decision for stimulus ‘A’. In few cases, a decision for stimulus ‘B’ is made because of the noise (purple reaction time distributions). (**D**) Experiment 1. In the psychophysical experiments, dominance is quantified as the percentage of responses which are in accordance with the first vernier. According to the one-stage model, vernier dominance increases when total stimulus duration increases (blue line), in stark contrast to the performance of human observers (green line; mean dominance across observers; error bars represent standard error of means, SEM). For the model, dominance is quantified as the percentage of trials in which the diffusion process hits the upper boundary (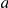). (**E**) Two-stage model. The input is first integrated, before it is buffered and fed as a constant drift into the drift-diffusion process. (**F**) The input is delayed by 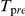 and integrated with a leak. The value of the first stage is read out after stimulus termination 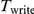, written into a buffer, and fed as a constant drift rate into the diffusion process at times greater than 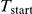. Longer input durations lead to stronger negative drifts. Hence, the probability to hit the lower boundary 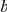 increases with increasing vernier durations. (**G**) Performance of the two-stage model (purple line) is similar to the performance of human observers for total durations up to 80 ms (green circles, same human data as in D).

If the ISI is 0 ms, i.e. the two verniers are presented in immediate succession, feature fusion occurs [36]. Observers perceive only one single vernier with a smaller offset because the vernier offsets integrate and partially cancel each other out (Figure 2A; [37], [38]). Our feature fusion experiments with Vernier stimuli are analogous to classic feature fusion experiments with color. For example, observers perceive one single yellow disk when a red disk is rapidly followed by a green disk [39].

### Dominance of the second stimulus

In experiment one, vernier stimulus ‘A’, offset either to the left or right, was immediately followed by a second vernier stimulus ‘B’ with opposite offset direction (right or left, respectively). The durations 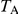 and 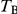 of both verniers were equal, i.e. 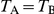, but varied from 10 to 80 ms, each. Vernier stimulus ‘B’ dominates the percept the stronger the longer both vernier stimuli ‘A’ and ‘B’ are presented (Figure 2D). For example, when the two vernier stimuli are presented for 20 ms each, observers report a percept corresponding to stimulus ‘B’ in 60% of the trials, while ‘A’ is reported in only 40% of the trials. When the two stimuli are presented for 40 ms each, observers report a percept corresponding to stimulus ‘B’ in 67% of the trials, while ‘A’ reported in only 33% of the trials.

We wondered whether the dominance of the second stimulus could be explained by classical noisy accumulator models, also called Drift-Diffusion models. In the standard, one-stage Drift-Diffusion Model [20], [22], [23], [27], evidence for ‘A’ or ‘B’ translates directly into the drift rate (upward for ‘A’, downward for ‘B’) of a decision variable 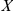 (Figures 2B, C). As usually, we added noise to the drift process leading to a random walk of the trajectory. The noise accounts for both noisiness of the evidence itself (an important aspect in the moving-dot paradigm [9], [33], [34], [40]) and internal noise in the brain. After presentation of both stimuli, the drift goes back to zero. A decision is made when 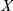 hits the upper (for ‘A’) or lower bound (for ‘B’).

In this one-stage model, dominance of stimulus ‘A’ is the stronger the longer the presentation times of ‘A’ and ‘B’, 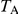 and 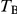 respectively. This is in striking contrast to the experimental results. We found that the qualitative nature of the results is independent of the specific choice of parameters of the one-stage drift diffusion model: for all tested parameters, the dominance of the second stimulus decreased with increasing duration (whereas the dominance of the second stimulus increased in the experiments). Whereas, for certain, fixed stimulus durations, we could achieve dominance of the second stimulus with specifically optimized parameters, we could never achieve dominance of the second stimulus for the entire range of stimulus durations with one set of parameters.

We explored whether minor modifications of the one-stage drift-diffusion model can explain the dominance of the second vernier. For example, we replaced the noisy accumulator by a noisy leaky accumulator. However, this did not change the results qualitatively. We then tested a very basic two stage model. During stimulus presentation, the stimulus served as the drift in a noisy leaky integrator model. After stimulus termination, the leak was artificially set to zero and the integration continued as a free, unbiased noisy diffusion process. In other words, the result of the leaky evidence integration served as initial condition for the leak-free diffusion process. While qualitatively such a drift-diffusion model explains the dominance results well (Supporting Figure S3B), we suggest an alternative model, which accounts very well for both the dominance and the reaction time distributions.

In this two-stage model, the evidence integration enters the second stage as a drift rate rather than as a bias in the initial condition. (a) During stage one, evidence integration is leaky and dominated by the intrinsic noise of the stimulus. The variable of noisy evidence integration is 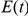. (b) Stage two starts after a fixed time 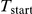 after stimulus onset and ends when a second variable 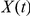 hits the upper or lower decision threshold. (c) The variable 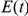 of the leaky integrator of stage one sets the drift in the (leak-free) drift-diffusion model of stage two.

The combination of (b) and (c) implies that, for long stimuli, stage two is a drift-diffusion model with time-dependent drift set by the momentary value 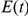 of the integration variable of stage one. In case that the total duration of the stimulus is shorter than the time needed to reach the decision threshold in stage two, the value of the leaky integrator of stage one at the end of the stimulus is written into a buffer and this buffered value serves, during the remaining time, as the (constant) drift for the diffusion process in stage two until a decision is reached. In the limit that stimuli are shorter than 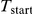, stage two has therefore a constant drift. In the limit that stimuli are presented for times much longer than 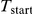 (so that 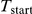 is negligibly short compared to the stimulation time), our two-stage model becomes equivalent to a standard one-stage drift-diffusion model with a time-dependent drift that is given by the low-pass filtered version of the input signal. However, for very short stimuli, the prediction of our two-stage model is remarkably different from that of a standard one-stage model – and these ultra-short stimuli are at the center of our study.

The results on stimulus dominance during the feature fusion paradigm with two short Verniers can indeed be explained by the two-stage model (Figures 2E, F). Since our stimuli are comparatively strong (over 90 percent accuracy for stimli presented separately), we consider the limit where the evidence integration in stage one is noise-free. Hence, in the first *integration stage*, evidence for stimulus ‘A’ and ‘B’ is simply accumulated in a noiseless forgetful (leaky) integrator (see also [30]). The time scale of forgetting is related to the time over which an ideal observer expects stimuli to remain constant (see Materials and Methods). The second phase, the *decision stage* starts at a fixed time 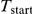 and consists of a standard drift-diffusion model without leak (Figure 2F, bottom panel). For a sequence of two short stimuli, the stimulation ends before 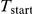 so that at the termination of the second stimulus (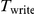), the output of the evidence integration is written into a buffer and fed later from the buffer as a constant drift rate into stage two. The two-stage model captures the dominance of the second vernier very well (Figure 2G).

### Reaction times

**Figure 3:**
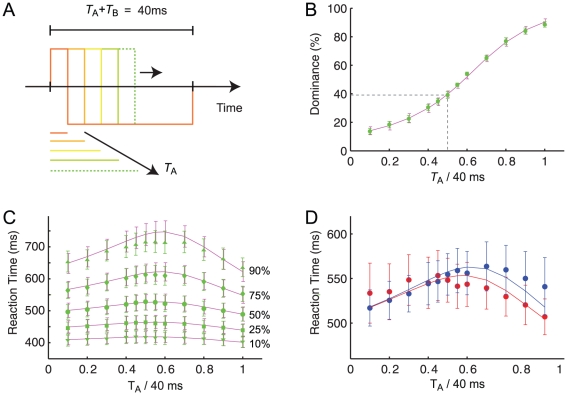
Experiment 2. (**A**) A vernier (stimulus ‘A’) of duration 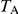 is followed by a second vernier with opposite offset direction (stimulus ‘B’) of duration 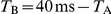. (**B**) The longer the first vernier stimulus is presented, the stronger is its dominance (green circles). If first and second verniers are of the same duration, the second vernier dominates performance, i.e. performance is below 50% (dashed line). Relative vernier duration, 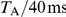, is plotted on the abscissa, mean vernier dominance across observers is plotted on the ordinate. The two-stage model (purple line) fits the psychophysical data well. (**C**) The 10% (downward pointing triangles) and 25% fastest responses (squares) vary only slightly with the relative vernier duration. The median (circles), the 75% quantile (diamonds) and 90% quantile show a strongly inverted U-shaped pattern (mean across observers). When either the first or the second vernier clearly dominate performance, response times are shorter than when first and second vernier are equally long (relative vernier duration 0.5). The two-stage model (purple lines) fits the psychophysical data well. (**D**) Mean response times across observers for the responses to the first vernier (red circles) and the second vernier (blue circles) show a similar pattern. The two-stage model captures this behavior well (solid lines). Error bars represent SEM.

The critical test for models of decision making is to account for reaction time *distributions* rather than accuracy [20]. We therefore wondered whether the two-stage model captures the reaction time distributions in the fusion experiments. In experiment two, stimulus ‘A’ (the first vernier stimulus) was presented for a duration 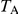, immediately followed by stimulus ‘B’ (a vernier with opposite offset) of duration 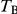 with 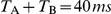 (Figure 3A). Parameters of the two-stage model were adapted individually for each observer and kept fixed across all stimulus conditions. The dominance of the first vernier stimulus increased when 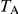 increased (Figure 3B). Reaction times for strongly biased situations (e.g. where the first vernier stimulus is much longer than the second one or vice versa) are faster (75% of decisions made before 560 ms) than those in conditions with dominance around 50% (75% of decisions made before 610 ms) leading to an inverted-U-shaped curve of the reaction time quantiles (Figure 3C). The same pattern is observed when responses for the first and second vernier stimulus are analyzed separately (Figure 3D).

**Figure 4:**
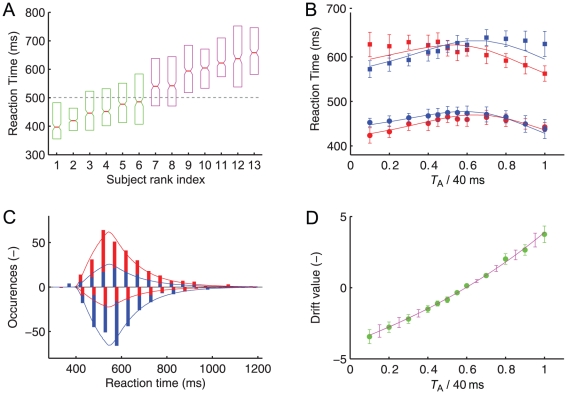
Experiment 2, continued. (**A**) Slow and fast responders in experiment 2. Box-plots of the reaction times for all 13 observers. A vernier stimulus was followed by a second vernier with opposite offset direction of 20 ms duration each. The lower and upper boundaries of the boxes represent the first and third quartile of the reaction time distribution. The median and its 95% confidence interval are indicated by the central line and the notch. Observers are ordered according to median response times. We separate observers in two groups. One group (green boxes) has median response times faster than 500 ms (dashed horizontal line), the other group slower than 500 ms (purple boxes). (**B**) Mean reaction time as a function of relative vernier duration for the first vernier (red symbols) and the second vernier (blue symbols) for fast responders (squares) and slow responders (circles). The solid lines represent the fit of the two-stage model. (**C**) Reaction time histograms of a typical observer showing responses to the first vernier (in red) and the second vernier (in blue). Responses are plotted for two stimulus conditions, where either the first vernier dominates (positive values; first vernier stimulus was presented for 32 ms followed by the second vernier of 8 ms) or the second vernier dominates (negative values; first vernier stimulus was presented for 8 ms followed by the second vernier of 32 ms). The solid lines are the corresponding two-stage model fits. The reaction time distributions for the other 12 observers are shown in the Supporting Figure S1. (**D**) The drift of the two-stage model (purple line) compared to the alternative two-stage model where the drift parameter was optimized for each stimulus condition independently (green circles). All other parameters are kept constant across different stimulus conditions but are different for each observer. Error bars represent SEM for both model variants.

Median response times varied strongly across the 13 observers (Figure 4A). We separated the observers into a group of fast responders (median reaction time <500 ms) and one of slow responders (median reaction time >500 ms). While the reaction times of both groups show an inverted U-shape function, the qualitative picture is different between slow and fast responders. If the first vernier stimulus is presented for a short time only, fast responders are particularly fast whereas slow responders are particularly slow. The two-stage model qualitatively reproduces this behavior (Figures 4B,C, Supporting Figure S1).

### Evidence integration in stage one as drift in stage two

For each stimulus condition, the outcome of the leaky integration in the first stage serves as a drift of the leakfree drift-diffusion model during the second stage of the two-stage model. For short stimuli, like the ones considered so far, where the stimulus ends before the integration, the result of stage one is written into a buffer and used as a constant value of the drift in the decision stage. In other words, the evidence at stimulus termination serves as *drift* value, rather than as an initial condition of stage two.

As an alternative, we have also analyzed a drift-diffusion model where the drift was taken as a free parameter, optimized for each stimulus condition independently so as to optimally predict the distribution of reaction times. The drift predicted from this model (which has more degrees of freedom) is statistically not different (as determined by a two way repeated measures analysis of variance) from our two-stage model where the drift is not a free parameter but the result of stage one. This finding suggests that the simple preprocessing by leaky integration correctly determines the drift rate (Figure 4D).

Further above we had reported that a qualitative fit of the dominance was possible by a noisy leaky integrator, if the leak was set to zero at the end of the stimulus. In such a model, the result of the leaky evidence integration serves as the *initial value* for a free diffusion process. The results of Figure 4D, however, indicate that the result of the leaky integration in the first stage should be used as the *drift*, and not as an initital condition for the diffusion in stage two. The results of Figure 4D can therefore be considered as a strong argument in favor of the two-stage model. In the following, we consider other aspects of the two-stage model.

### Writing into the buffer

If the writing into the buffer is triggered at stimulus termination, as assumed in the two-stage model, the question arises why the switch from ‘B’ to the background, but not that from stimulus ‘A’ to ‘B’, triggers the transition from stage one to stage two in the two-stage model. We suggest that the large change from a vernier stimulus to background is “interpreted” as stimulus termination because there is a strong neural off-transient for a change from ‘A’ to a blank screen, whereas there are no on- and off-transients for a change from ‘A’ to ‘B’, respectively [41]. This is well in accordance with a Bayesian approach (see Supporting Figure S2) suggesting that feature integration should terminate when it becomes unlikely that the momentary stimulus is a continuation of the previous stimulus. The readout in the two-stage model should therefore start when a novelty value of the momentary stimulus crosses a predetermined threshold (cf. Supporting Text S1).

We tested this prediction by the psychophysical experiment in Figure 1, where the first vernier stimulus was followed by a blank background (interstimulus interval; ISI) before the second vernier was presented. With an ISI of 20 ms, the two vernier stimuli, presented for 10 ms each, became individually discriminable. Observers could tell whether the first stimulus was offset to the left or to the right by motion cues [41], [42]. However, for a sequence of ‘A’ immediately followed by ‘B’ with 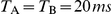, verniers are not individually visible even though the total duration is 40 ms as in the sequence with the 20 ms ISI. This suggests that in the condition with the 20 ms ISI the termination signal of the first vernier stimulus stopped evidence integration and wrote the result into a buffer, for later use in stage two, whereas evidence was integrated across the two vernier stimuli in the experiment without the blank, before the final result was written into a buffer.

### Start of the drift-diffusion process

In our experiments with ultra-short stimuli, the time 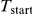 where the read-out from the buffer starts, occurs after stimulus termination (and is included in the non-decisional time 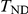. We also tested a model where the decision process was triggered at stimulus termination, i.e., at the same moment when the result of evidence integration is written into the buffer (i.e. 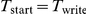). Such a model predicts that reaction times increase with total stimulus duration (data not shown), which disagrees with our observation that, for a given level of dominance, the mean reaction times remain largely constant for total stimulus durations of 20 ms, 40 ms, and 80 ms (Supporting Figure S3 D).

### Two-stage model in the case of longer stimuli

For most of the stimuli considered so far, the total stimulus duration was below 40 ms. In this case, the two stages of the model are sequential and do not overlap. However, for longer stimuli, evidence integration of stage one is not finished at the moment of 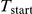 when the diffusive decision process in stage two is started.

Indeed, a model with *fixed* drift in stage two works well for stimuli up to a total duration of 80 ms, but breaks down at 160 ms (data not shown). However, our two stage model assumes that as soon as stimuli extend beyond 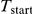, the momentary value of the evidence integration stage is written into the buffer and immediately used as drift in the diffusion process of stage two. The drift is updated continuously so that the diffusion process becomes time-varying. The fact that a constant drift in stage two fails when the stimulus extends over 160 ms indicates that the parameter 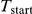 of our model is much shorter than 160 ms. We tested this by fitting 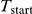 for individual subjects such that the mean square error in the dominance was minimized across all stimulus durations, including the 160 ms conditions. The optimal values for 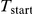 were indeed smaller than 160 ms (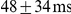, 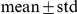, 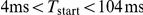).

In the model, we explored the situation that the first stimulus becomes much longer than 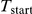. Obviously, if the first stimulus is made very long, our two-stage model then predicts that the first stimulus dominates.

## Discussion

Most models of decision making do not account for the timing of stimuli. Likewise, most experiments use long stimulus durations in the range of several hundreds of milliseconds to seconds [9], [31], [32] or constant stimuli [20], [22], [25]. However, decision making has to occur in many situations in less than 100 ms, for example, when driving a car or playing soccer. Here, we have shown that rapid decision processes show very different characteristics than decision processes on longer time scales. For example for short stimulus durations, later presented stimuli dominate over stimuli presented earlier. We propose that these processes are also present in longer lasting decision processes, but are hidden and barely measureable.

To study the dynamics of rapid decision processes, we used a feature fusion paradigm. This paradigm relies on the well known effect of visual integration masking [43], [44] and follows partly Bloch's law [45]. The results of our experiments are in agreement with earlier results on feature fusion [38] and backward masking experiments [43], but do not agree with the traditional *one-stage models* of decision making in which evidence is integrated until a decision boundary is reached. The results of our experiment rather support a *two-stage model* in which evidence integration is separate from the actual decision process. This model is fundamentally different from classical drift-diffusion models [20], [22], [23], [27], race models [18], [19], [25], [32], attractor models [10], [30], [31], one-stage models with pre-processing [28], and “parallel” two-stage models [21], [46]. All these models predict the first stimulus to dominate when 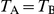 in contrast to the fusion results.

### The two-stage model

In our model, we assumed several components which are worth discussion each. First, evidence integration in stage one must be leaky. It is the leak that explains why, when the first and second vernier stimulus are of the same duration (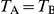), the second vernier stimulus dominates (experiment one). The leak in our model arises naturally from a Bayesian approach and can be traced back to the fact that stimuli are expected to change in natural environments. Similar to our Bayesian novelty detection approach (cf. Supporting Text S1), the leaky evidence integration can also be derived in the framework of Kalman filters [47]–[50].

Second, the accumulated evidence must transferred at an appropriate moment and written into a temporal buffer. Such a buffer is necessary since decisions often occur a considerable time after the stimulus has disappeared. We suggest that the precise moment of transfer 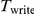 is set by a novelty score monitored during evidence integration (see Supporting Text S1). Such a novelty signal and subsequent buffering explains why the two vernier stimuli are perceived individually, if the stimuli are separated by a blank screen (ISI), but fused into a single percept in the absence of the blank. In this sense, feature fusion can be interpreted as a failure to detect the onset of a new stimulus because the new evidence is not sufficiently different to raise a ‘novelty signal’. In contrast, the switch from stimulus to background creates a sufficiently strong transient to stop the feature integration process (Supporting Figure S2).

Third, the noisy decision process is triggered at a fixed time 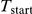 after stimulus onset. If the decision process were triggered at 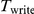, reaction times would increase with stimulus duration. This is, however, not the case (Supporting Figure S3D). From the fact that our model assumes a fixed start time of the second stage, it necessarily follows that we have to distinguish two different situations: If the total stimulus duration is shorter than 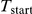, we need to bridge the time between the end of the stimulus and decision by storing the intermediate result of evidence integration into a buffer. This value is then used in stage two as a fixed mean drift rate. If the total stimulus duration is longer than 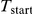, the result of stage one is used online as a time-dependent drift for all times 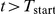 until the end of the stimulus (at which point it is again ‘frozen’ and transferred into the drift-buffer.).

Our two stage model is similar to previous two stage models in which sensory processing, e.g. motion processing or contrast detection, precedes a decision making stage (e.g. [15], [16], [46]). In our model, the sensory integration stage is leaky to account for the dominance of the second vernier.

### Leakage

Our two-stage model comprises a leaky integration stage followed by a drift-diffusion stage. The question arises whether or not a one-stage model with leak in the drift-diffusion process can explain the results. However, this is not the case because in such a model always the first stimulus dominates because the leak pushes the decision variable 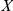 towards the starting point and not across it (Supporting Figure S3).

Another way to integrate the leak into a one-stage model is to directly transform the input by a leaky integrator (like our stage one) and to use the outcome of the leaky integrator as a time-variant drift in stage-two (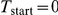, 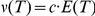). However, using stage one only for pre-processing will not change the pattern of results [28]. In such models, the decision variable also moves towards the decision bound for stimulus ‘A’ before dropping back to chance level. Therefore, these models also show a dominance of the first stimulus.

### Window of integration

The novel features of the two-stage models are observable well only for stimuli in the range of up to about 100 ms. This duration is in line with the duration of visual integration found in other studies [51]–[53]. One of the paradoxical aspects of our model is that the second stage starts at a fixed time 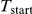. Obviously, if the duration of a stimulus extends beyond 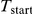, then the stage of evidence integration and that of stochastic decision making (stage two) will overlap and the separation into two distinct phases disappears (see Supporting Text S1). Therefore it is not surprising that for longer stimulus durations standard one-stage models work well [27], [29], [54].

### External and internal noise

In our model, a deterministic filter (leaky integrator) is applied in stage one to a step-like input, representing a noiseless stimulus. This is the limiting case where the stimulus is considered to be of high contrast. In a more realistic scenario the stimulus itself is noisy. The stochasticity of stimuli leads, after stage one, to a noisy result of evidence integration, which is written into the buffer and then used as drift for stage two. This noisy result is modeled by the variance of the drift constant of stage two. It is therefore tempting to relate the stochasticity of drift constants to sensory or physical noise. The stochasticity of stage two may be related to internal noise in the brain [32], [55]. What is the advantage of adding a separate noisy decision process? It is well known that human observers can manipulate the speed-accuracy trade-off according to instruction or reward scheme by a change in strategy corresponding to a shift of the initial condition, 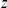, or the decision thresholds in the drift-diffusion process [13], [54].

### Accumulators in decision making and motor preparation

Neurons in the superior colliculus [56], the LIP [9], [32], [33], the pre-motor cortex [57], [58], and the dorsoventral lateral prefrontal cortex [11], [12], [59] were shown to be involved in decision making. The firing rate of these neurons increases as long as stimuli are displayed. This ramping activity may relate either to evidence accumulation (“stage one”) or to decision making (“stage two”). Future experiments with feature fusion stimuli may be used to decide between these two alternatives.

### Summary

In summary, it is often (intuitively) assumed that visual input directly translates into decisions. A stimulus presented first should drive decisions stronger and faster than a later stimulus (first in, first out). This is obviously correct when the two stimuli are long, because a decision may be reached even before the second stimulus can influence decision masking. In this case, we can assume that evidence integration and decision making are the same. However, for short stimuli this is not the case. Evidence integration and decision making can only be disentangled, when the two stimulus alternatives are presented within one trial (feature fusion) but not when only one stimulus is presented per trial, as it is usually. The distinction between evidence integration and decision making is described well by our two-stage model, where rapid stimuli are integrated and buffered before the decision process starts.

## Materials and Methods

### Ethics statement

All participants signed informed written consent. The study was approved by the Commission cantonale (VD) d'éthique de la recherche sur l'être humain (Lausanne, Switzerland) and conducted according to the principles expressed in the Declaration of Helsinki.

### Observers

A total of 24 observers (8 female, aged 21–32 years) signed informed written consent. Participants had normal or corrected-to-normal visual acuity as measured by the Freiburg visual acuity test [60]. All but two observers (the first and second author) were naive to the purpose of the study. Naive observers were paid students from local universities.

### Setup

Stimuli were presented on a Tektronix 608 X-Y display or a HP 1332A X-Y display. Both X-Y displays were equipped with a P11 phosphor and controlled by a PC via a fast 16 bit DA converter. Stimuli were presented at 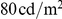, a 1 MHz dot rate, a 500 Hz refresh rate, and a dot pitch of 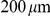. Viewing distance was 2 m. The room was dimly illuminated by a background light (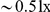) to prevent adaptation to scotopic vision. Stimulus contrast was close to 1.0. In each experiment, the conditions have been presented randomly interleaved to reduce the influence of hysteresis, learning, or fatigue in the averaged data.

### Stimuli

The vernier stimuli were composed of two vertical segments. Each segment was 10′ (arc min) long, 0.5′ wide, separated by a vertical gap of 1′. A small horizontal offset was inserted between the upper and the lower segments (Figure 2A). Horizontal offset sizes ranged from 30″ to 40″ (arc sec). Offsets were chosen individually to be at least twice the offset size of the offset discrimination threshold for a single vernier stimulus of 20 ms duration as determined using the adaptive PEST procedure [61]. A sequence of two vernier stimuli with opposite offset directions was presented foveally in rapid succession. The offset direction of the first vernier (stimulus ‘A’) was chosen randomly in each trial (left or right). The second vernier (stimulus ‘B’) had an offset direction opposite to that of the first vernier. If, for example, the first vernier stimulus was offset to the left, the second vernier was offset to the right, and vice versa. Observers perceived only one fused vernier and were asked to report the position of the lower segment with respect to that of the upper segment by pressing one of two push buttons. Observers were instructed to respond as rapidly as possible, but also as accurately as possible. No feedback about performance was given. Naive observers did not know that a sequence of two vernier stimuli was presented.

### Performance measure

We computed dominance, defined as the proportion of trials on which the response matched the offset direction of the first vernier stimulus. Thus, values above 50% indicate dominance of the first vernier (stimulus ‘A’); values below 50% indicate dominance of the second vernier (stimulus ‘B’). 50% vernier dominance is the point of subjective equality, i.e. first and second vernier stimulus equally contribute to performance.

### Experiment 1

First vernier stimulus (‘A’) and second vernier (‘B’) were presented in immediate succession (Figure 2). Both vernier stimuli had either the same duration or the duration of one of the verniers was four times longer than the other. The total duration of the first and second vernier was 20 ms, 40 ms, 80 ms, or 160 ms. All conditions were presented in a random order. Every condition has been repeated 400 times per observer.

### Experiment 2

As Experiment 1, except for that the duration 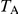 of the first vernier was varied in 12 steps between 0 ms and 40 ms. The total duration 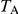
+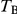 always summed up to a total of 40 ms (Figure 3A). Every condition has been repeated 400 times per observer.

### Reference experiment

In Figure 1, an ISI was inserted between the first and second vernier stimulus. Observers were informed about the experimental design and asked to indicate whether the first or second vernier stimulus was offset to the right.

### Reaction time analysis

Reaction times below 300 ms or above 1200 ms were excluded from analysis to reduce the impact of motor errors and unattended trials (less than 3% of the trials).

### Model

We model the stimuli by a time-varying input signal 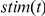, which is +1 during the presentation of stimulus ‘A’, −1 for stimulus ‘B’ and 0 otherwise. In the evidence accumulation stage of the two-stage model, the stimulus is subjected to leaky integration: 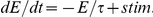 Since our stimuli have high contrast, the evidence integration is modeled as a noise-free process.

For times larger than 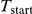 the integrated evidence 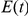 is fed as the drift into the noisy drift-diffusion model at stage two. We distinguish two different cases. a) Stimuli are shorter than 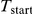. At the termination of stimulus ‘B’ (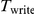) the integrated evidence 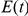 is stored and written into a buffer. Later, for 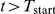 the buffered value is used as the mean drift rate 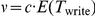 with a fixed scaling factor 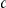 for the decision stage, which encompasses a standard drift-diffusion model. b) Stimuli are longer than 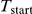. In this case the momentary evidence 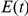 is used as the mean drift 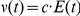 for 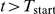. Again, at the end of the stimulus, the last value of the evidence is buffered and used as drift henceforth.

During stage two, in every trial, a decision variable 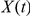 is initialized at 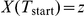 and evolves according to the Langevin equation 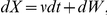 where 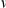 is the drift rate and 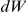 is a Wiener process, which introduces noise to the decision process. A decision is made when the decision variable 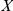 reaches one of two decision boundaries 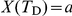 (decision ‘A’) or 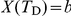 (decision ‘B’). The associated reaction time is the sum of a non-decisional time 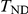 (which accounts for sensory delay 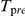, and the evidence integration and buffering times as well as motor delays) and the time 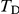 when the decision variable 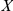 reaches the boundary. We used the Ratcliff extension [20] of a standard drift diffusion model, in which the non-decisional time 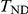, the initial condition 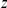 and the drift rate 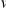 vary stochastically from trial to trial. The non-decisional time 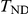 is drawn from uniform distributions with mean 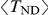 and width 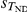. The initial condition 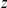 is drawn from uniform distributions with mean 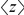 and width 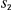. The drift rate 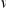 is drawn from a Gaussian distribution with mean 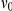 – the output of the first stage – and standard deviation 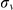. 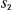 and 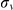 represent noise in the evidence accumulation.

As a reference, we used a one-stage model, which encompasses a standard drift diffusion model, in which the drift rate depends on time and is given by the input signal: 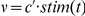. In this model, the drift becomes zero after the end of the stimulus. We also simulated leaky variants of this one-stage model, for details see Supporting Text S1.

### Fitting

In the first step, the parameters 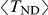, 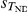, 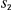, 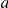, 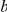, 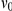 and 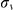 of the decision stage were fitted to the experimentally obtained cumulative reaction time distributions by minimizing the product of the p-values of the Kolmogorov-Smirnov statistic for each stimulus condition [62], [63]. Responses to stimuli ‘A’ and ‘B’ and different stimulus conditions were fitted simultaneously using the fast-dm software of Voss & Voss [64]. For both experiments, fits were done individually for each observer. In the experiment of Figures 2, all parameters except the mean drift rate 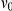 and the drift variability 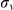 were the same in all stimulus conditions. The drift was calculated from stage one. Drift variability was a function of stimulus duration. In the experiment of Figures 3 and 4, only the mean drift rate was varied across conditions and calculated from stage one. In order to obtain the parameters 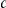 and 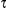 of the evidence integration in stage one we ran a simulation experiment with free drift rates as in Figure 4D. The obtained mean drift rates 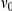 were then used to fit the time constant 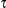 and the scaling factor 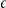, again separately for each observer. This fit was done using the fit-routine of MATLAB. Finally, to extract the optimal values for 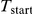, we first used the data of experiment 1 with stimulus durations 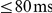 and fitted the parameters of both stages with the described procedure. Then, we performed a line scan of all values of 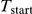 and identified the value that minimized the mean square error of the measured dominance, now including the long duration of 160 ms.

Parameters are different for each observer, i.e. 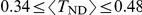, 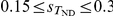, 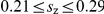, 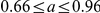, 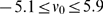, 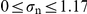, 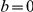, 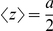 and for stage one 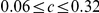 and 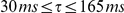.

## Supporting Information

Figure S1Reaction time histograms of 12 observers for responses to the first vernier (red) and to the second vernier (blue). Responses are plotted for the two stimulus conditions 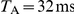 and 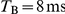 (positive values) and with 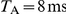 and 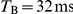 (negative values). The solid lines are the corresponding two-stage model fits. The Kolmagorov-Smirnov (KS) statistic for each fit is given.(PDF)

Figure S2Bayesian model of feature fusion. **A–C.** Stimulus ‘A’ (red bar) and ‘B’ (blue bar) are presented with durations of 10 ms (A), 20 ms (B), or 40 ms each (C). The upper panel of each subplot shows the posterior probability (belief) as a function of time (*A* - red curve, *B* - blue curve, *blank* - black curve). The lower panels show the novelty signal 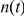, which triggers the decision process in the two-stage model. The dashed line indicates the background novelty 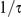. No novelty signal is generated by a direct transition from ‘A’ to ‘B’. Only the onset of ‘A’ and the termination of ‘B’ generate novelty signals (A–C). The posterior at the end of stimulus ‘B’ shows a preference for *B*, which increases with increasing stimulus duration. **D.** The insertion of a *blank* of 20 ms between ‘A’ and ‘B’ generates additional novelty signals at the termination of ‘A’ and the onset of ‘B’. The *blank* prevents feature fusion of ‘A’ and ‘B’: Stimulus ‘A’ has no influence on the “interpretation” of ‘B’.(PDF)

Figure S3Leaky drift diffusion model and behavior of two-stage model for long stimuli. (**A**) Vernier dominance as a function of total stimulus duration in a one-stage drift-diffusion model with leak (for details see Supporting Text S1). The stimulus strength (i.e. the magnitude 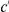 of the drift rate) is varied from 0.0 (chance level, dashed green) to 10 (orange line) in steps of 2.5. The dominance of the first stimulus increases with total stimulus duration for all drift rates different from 0.0 (no drift). (**B**) Dominance as a function of total stimulus duration, as in B, but for a leaky one-stage drift-diffusion model, in which the drift is switched off at the end of the stimulus. The model shows a dominance of the second stimulus for intermediate stimulus durations, which converts into a dominance of the first for long stimulus durations. (**C**) Dominance for the two-stage model (purple lines), compared with the results of experiment one (green lines). The model captures the results well and predicts increasing dominance for long total stimulus durations. (**D**) Mean reaction time corresponding to the experiment described in (A). Trials in which observers responded for the first vernier stimulus ‘A’ (red symbols) or stimulus ‘B’ have similar reaction times, if the total stimulus duration 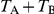 is 20 ms, 40 ms, or 80 ms. For a total duration of 160 ms, trials where observers decide for the first vernier stimulus are faster than those where they decide for the second vernier. The two-stage model (solid lines) captures response times for short stimuli well, but fails to predict reaction times for total durations of 160 ms. Error bars represent SEM.(PDF)

Text S1 **Technical Description of Alternative Models.**
(PDF)
