# Supplementary figures and images for: Paradoxical Evidence Integration in Rapid Decision Processes
Source: PLoS Comput Biol. 2012 Feb 16;8(2):e1002382. doi: 10.1371/journal.pcbi.1002382 (PMC3280955; doi:10.1371/journal.pcbi.1002382)

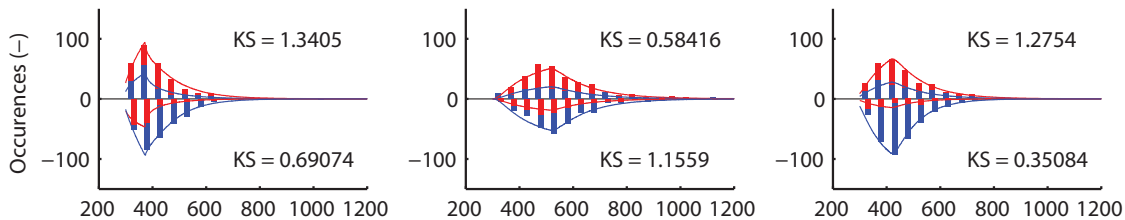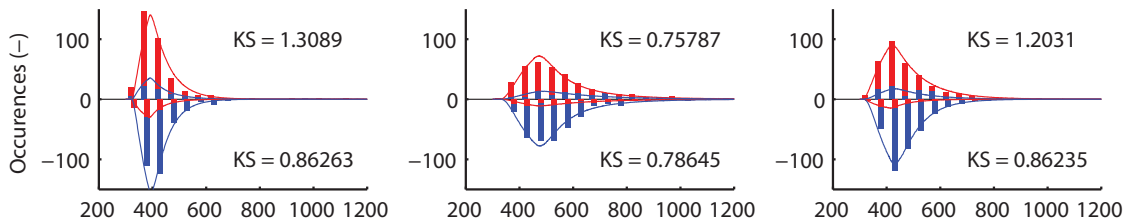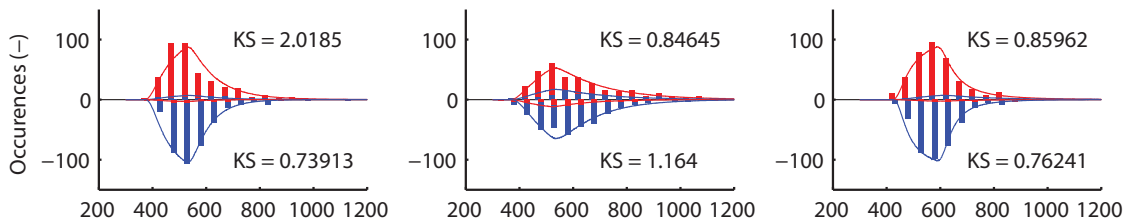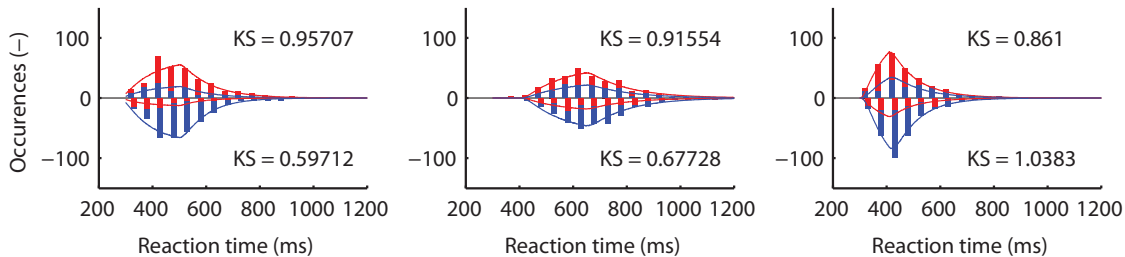

Supplement: Figure S1 — Reaction time histograms of 12 observers for responses to the first vernier (red) and to the second vernier (blue). Responses are plotted for the two stimulus conditions and (positive values) and with and (negative values). The solid lines are the corresponding two-stage model fits. The Kolmagorov-Smirnov (KS) statistic for each fit is given. (PDF) [file pcbi.1002382.s001.pdf]

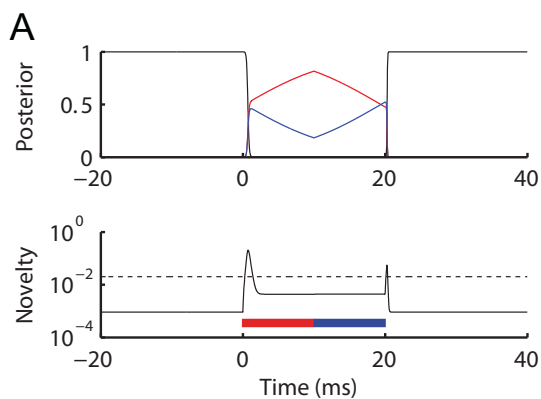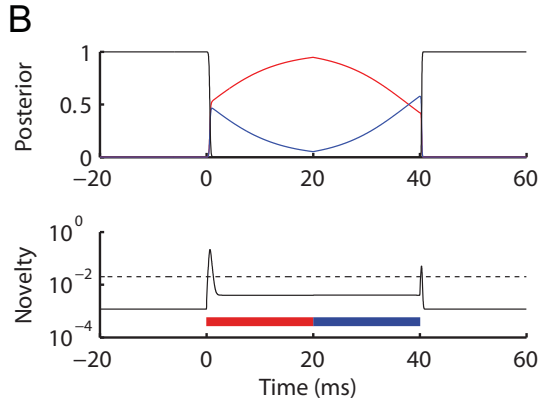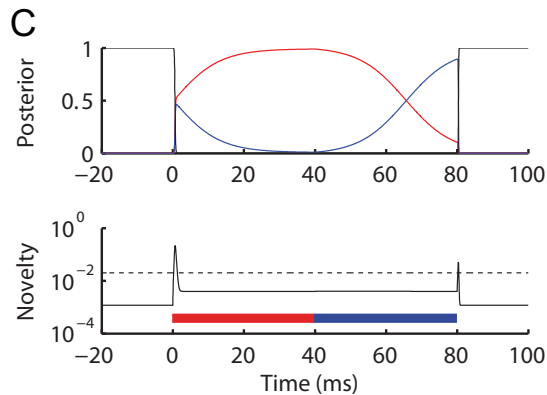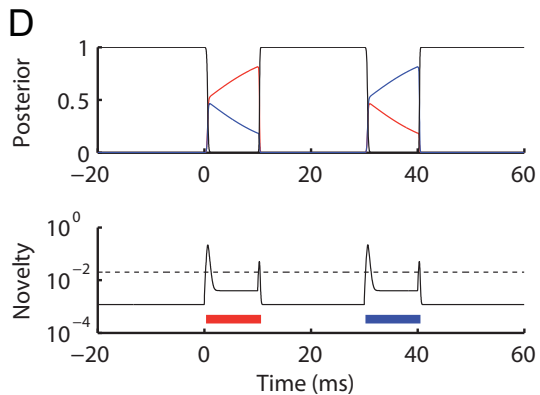

Supplement: Figure S2 — Bayesian model of feature fusion. A–C. Stimulus ‘A’ (red bar) and ‘B’ (blue bar) are presented with durations of 10 ms (A), 20 ms (B), or 40 ms each (C). The upper panel of each subplot shows the posterior probability (belief) as a function of time (A - red curve, B - blue curve, blank - black curve). The lower panels show the novelty signal , which triggers the decision process in the two-stage model. The dashed line indicates the background novelty . No novelty signal is generated by a direct transition from ‘A’ to ‘B’. Only the onset of ‘A’ and the termination of ‘B’ generate novelty signals (A–C). The posterior at the end of stimulus ‘B’ shows a preference for B, which increases with increasing stimulus duration. D. The insertion of a blank of 20 ms between ‘A’ and ‘B’ generates additional novelty signals at the termination of ‘A’ and the onset of ‘B’. The blank prevents feature fusion of ‘A’ and ‘B’: Stimulus ‘A’ has no influence on the “interpretation” of ‘B’. (PDF) [file pcbi.1002382.s002.pdf]

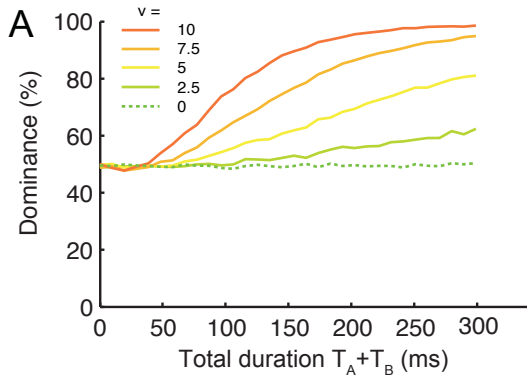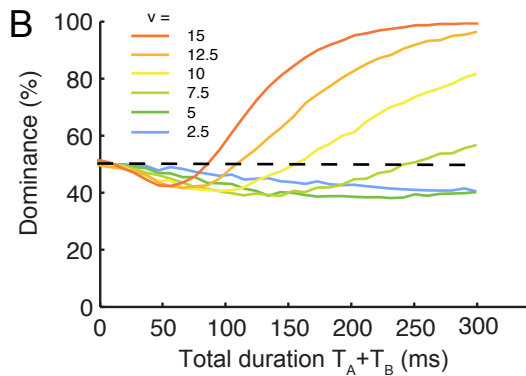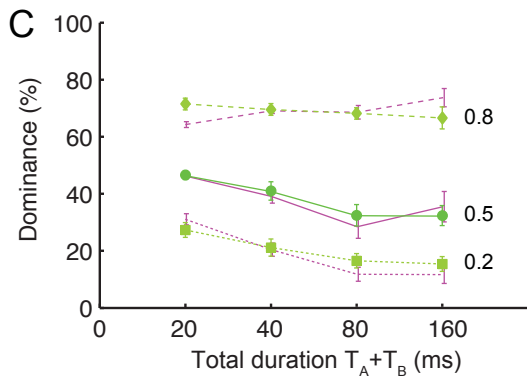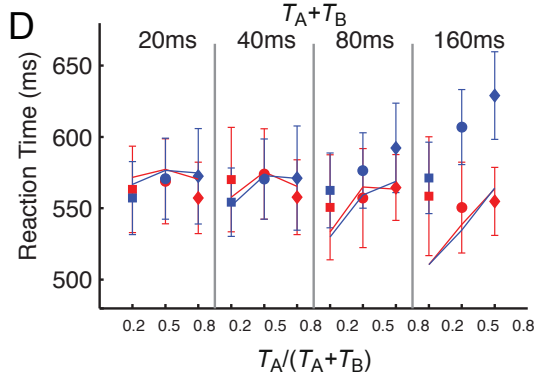

Supplement: Figure S3 — Leaky drift diffusion model and behavior of two-stage model for long stimuli. (A) Vernier dominance as a function of total stimulus duration in a one-stage drift-diffusion model with leak (for details see Supporting Text S1). The stimulus strength (i.e. the magnitude of the drift rate) is varied from 0.0 (chance level, dashed green) to 10 (orange line) in steps of 2.5. The dominance of the first stimulus increases with total stimulus duration for all drift rates different from 0.0 (no drift). (B) Dominance as a function of total stimulus duration, as in B, but for a leaky one-stage drift-diffusion model, in which the drift is switched off at the end of the stimulus. The model shows a dominance of the second stimulus for intermediate stimulus durations, which converts into a dominance of the first for long stimulus durations. (C) Dominance for the two-stage model (purple lines), compared with the results of experiment one (green lines). The model captures the results well and predicts increasing dominance for long total stimulus durations. (D) Mean reaction time corresponding to the experiment described in (A). Trials in which observers responded for the first vernier stimulus ‘A’ (red symbols) or stimulus ‘B’ have similar reaction times, if the total stimulus duration is 20 ms, 40 ms, or 80 ms. For a total duration of 160 ms, trials where observers decide for the first vernier stimulus are faster than those where they decide for the second vernier. The two-stage model (solid lines) captures response times for short stimuli well, but fails to predict reaction times for total durations of 160 ms. Error bars represent SEM. (PDF) [file pcbi.1002382.s003.pdf]
