# Supplementary material for: Paradoxical Evidence Integration in Rapid Decision Processes
Source: PLoS Comput Biol. 2012 Feb 16;8(2):e1002382. doi: 10.1371/journal.pcbi.1002382 (PMC3280955; doi:10.1371/journal.pcbi.1002382)
Supplement: Text S1 — Technical Description of Alternative Models. (PDF) [file pcbi.1002382.s004.pdf]

Paradoxical evidence integration in rapid decision processes - Supporting Text S1

Johannes Rüter[1,*], Nicolas Marcille[2], Henning Sprekeler[2], Wulfram Gerstner[2] and Michael H. Herzog[1]

1 Laboratory of Psychophysics, Brain Mind Institute, École Polytechnique Fédérale de Lausanne, 1015 Lausanne EPFL, Switzerland

2 Laboratory of Computational Neuroscience, Brain Mind Institute and School of Computer and Communication Sciences, École Polytechnique Fédérale de Lausanne, 1015 Lausanne EPFL, Switzerland

∗ E-mail: johannes.rueter@gmail.com;

Alternative Models

Novelty and leaky integration in a Bayesian model

The two-stage model of evidence integration has two central features: (a) evidence undergoes a leaky integration and (b) evidence values are read out and written into a buffer at the offset of stimulus 'B', but not after stimulus 'A'. These features can be motivated by a Bayesian model, which (a) shows that leaky evidence integration is a natural consequence when taking changing stimulus identity into account and (b) generates novelty signals with suitable properties to trigger the transfer into the buffer.

Suppose that an observer is looking at a screen that shows a stimulus whose identity (I) may change over time. At each time t, the stimulus induces a signal S_t in the visual system of the observer. The task of the observer is to infer the identity of the stimulus.

We describe the perception process within a probabilistic framework, i.e. we represent the current belief of the observer about stimulus identity by the probability $p(I;t)$ that the signal S_t

at time t is caused by a stimulus I. At stimulus onset ($t = 0$) the observer has no information about its identity. All stimuli are equally likely, i.e. $p(I; t = 0) = p_{\text{flat}}$ is flat. As time progresses, the observer gradually acquires knowledge on stimulus identity by integrating the evidence in the signals. The posterior probability $p(I; t)$ of the stimulus identity given the signal S_t at time t is

$$p(I; t) = \frac{p(S_t|I)\tilde{p}(I; t)}{\sum_I p(S_t|I)\tilde{p}(I; t)} , \tag{1}$$

where $\tilde{p}(I; t)$ is a prior probability and $p(S_t|I)$ is the likelihood of the signal S_t given stimulus identity I. The likelihood $p(S_t|I)$ represents the model of the environment that the observer has acquired through previous experiences.

If we used in Eq. (1) the posterior $p(I; t-\Delta t)$ at the last time step as a prior for calculating the posterior at time t (i.e. $\tilde{p}(I; t) = p(I; t-\Delta t)$), the observer would perform a lossless integration of information over time. Such a full temporal integration, however, makes the implicit assumption that the stimulus identity remains constant at all times. In the face of changing stimuli, the observer would interpret different stimuli as one and thus come to erroneous conclusions. To account for changes in stimulus identity, we introduce an additional stochastic variable $N \in \{new, old\}$, which signals if the current signal is "old", i.e. if it corresponds to the current belief so that the observer should continue the integration, or if it is "new". In the latter case, a prior $\tilde{p}(I; t) = p(I; t - \Delta t)$ that reflects previous evidence is not appropriate and should be replaced by the flat prior p_{flat}. This leads to a combined model

$$\begin{aligned} \tilde{p}(I; t) = p(I|new)p(new) + p(I|old)p(old) = \\ p_{\text{flat}}(I)p(new) + p(I; t - \Delta t)p(old) \, . \end{aligned} \tag{2}$$

Before we specify how $p(new)$ is calculated, let us first show that this model introduces an information leak, similar to the low-pass properties found in the integration stage of the two-stage model.

The leaky evidence integrator. Until now, we assumed that signals arrive in the visual system at discrete moments in time. Let us now consider the limit in which stimuli are presented in continuous time: $\Delta t \to 0$. To keep the amount of information in the signals finite as $\Delta t \to 0$, the amount of information per time bin Δt has to go to zero, i.e. the observer's model $P(S_t|I)$ has to become progressively less informative. The limit can be taken by using

$$P(S_t|I) = \frac{1}{N}\big[1 + W(S_t|I)\Delta t\big],$$

where N is the number of possible signals and $W(S_t|I)$ denotes an evidence rate, which is constant as $\Delta t \to 0$. The continuous dynamics of the posterior $p(I;t)$ can be derived by expanding Eqs. (1) and (2) in orders of Δt and disregarding all terms of order $(\Delta t)^2$. Taking the limit $\Delta t \to 0$ yields a nonlinear differential equation for the posterior:

$$\begin{gathered}\frac{\mathrm{d}}{\mathrm{d}t}p(I;t) = -n(t)\left(p(I) - p_{\text{flat}}(I)\right) + \\ W(S_t|I)p(I;t) - p(I;t)\sum_{I'} W(S_t|I')p(I';t)\,. \end{gathered} \tag{3}$$

Note that if it were not for the signal, the posterior $p(I;t)$ would relax towards the flat prior p_{flat} with a time constant $\tau(t) = 1/n(t)$. We denote the relaxation rate $n(t)$ as the *novelty*, because it is related to the probability $p(new)$ of the signal being new: $n(t) = \lim_{\Delta t \to 0} p(new)/\Delta t$. Since the signals become less informative as $\Delta t \to 0$, the probability $p(new)$ decreases with Δt such that the novelty $n(t)$ is well defined.

There are different approaches to calculating $p(new)$. In the absence of information on the stimuli, we can assume that there is a characteristic time τ after which the observer typically expects stimulus identity to change. The probability of a change within a short time window Δt is then given by $p(new) = \Delta t/\tau$ and the novelty $n(t) = 1/\tau$ is constant. This leads to a

leaky evidence integration with a constant leak time constant τ, quite similar to the evidence integration model in the main article (see stage 1 in the two-stage model in Figure 2F). Note that for constant $p(new)$, our model is equivalent to a hidden Markov model.

Novelty. A more elaborate approach to calculating $p(new)$ is to use the likelihood to calculate the probability that the current signal is in agreement with the current belief $p(I; t - \Delta t)$ of the observer. To this end, we again use the Bayesian approach and calculate the posterior

$$p(new|S_t) = p(S_t|new)\tilde{p}(new)/p(S_t),$$

where $\tilde{p}(new)$ is a prior probability that the signal is new and $p(S_t|new)$ and $p(S_t)$ are calculated using the probabilistic model:

$$\begin{aligned}
p(S_t|new) &= \sum_I p(S_t|I)p(I|new) = \sum_I p(S_t|I)p_{\text{flat}}(I) \\
p(S_t|old) &= \sum_I p(S_t|I)p(I|old) = \sum_I p(S_t|I)p(I; t - \Delta t) \\
p(S_t) &= p(S_t|new)\tilde{p}(new) + p(S_t|old)(1 - \tilde{p}(new))\,.
\end{aligned}$$

To fully specify this model, we have to choose a prior $\tilde{p}(new)$. Similar to the arguments in Eqs. (2) and (3), we allow the observer to accumulate evidence on the novelty of the signal over a given time interval τ_{new}. To this end, we again use a mixed prior $\tilde{p}(new)$ containing the old posterior $p(new|S_{t-\Delta t})$ and a constant prior $p_0(new) = \Delta t/\tau$. By taking the limit of continuous time, we obtain a differential equation for the novelty $n(t)$ at time t:

$$\frac{\mathrm{d}}{\mathrm{d}t}n(t) = -\frac{1}{\tau_{\text{new}}}\left(n(t) - \frac{1}{\tau}\right) + \left[W(S_t|new) - W(S_t|old)\right]n(t)\,,$$

where $W(S_t|new)$ and $W(S_t|old)$ are defined by

$$W(S_t|new) := \sum_I W(S_t|I) p_{\text{flat}}(I)$$

and

$$W(S_t|old) := \sum_I W(S_t|I) p(I;t) \,.$$

In essence, this model tracks the novelty by comparing the stimuli within a time window of duration τ_{new} with the current beliefs $p(I;t)$ for the different stimuli I. As long as belief and signal are in agreement, $W(S_t|new) < W(S_t|old)$ so that the novelty $n(t)$ remains smaller than $1/\tau$. Consequently, the leak term in Eq. (3) is small and information is integrated over a long time scale. When stimulus identity changes, there is a brief period in which the signals disagree with the current belief: $W(S_t|new) > W(S_t|old)$. As a result, the novelty $n(t)$ increases and $p(I;t)$ relaxes more quickly towards the flat prior – previous evidence is forgotten.

Simulations. We simulated the case of 3 different stimulus identities (stimulus A, stimulus B and *blank*) and 3 different signals ('A', 'B' and 'blank'). The fact that A and B are similar is modeled by a relatively small difference in the evidence rates $W(S_t|I)$ for stimuli A and B. In contrast, the evidence rate $W(S_t|I = blank)$ for *blank* vs. stimulus A or B is relatively high. The evidence rates $W(S_t|I)$ are given in Table 1. The time constants for novelty detection and expected stimulus identity change are $\tau_{new} = 1\,\text{ms}$ and $\tau = 50\,\text{ms}$. We used a time discretization of $\Delta t = 0.01\,\text{ms}$, which is sufficiently small to ensure that the discretization has no influence on the results.

The model can reproduce the central features of evidence integration in the psychophysical experiments (Figure 2G) and generates novelty signals needed for the transfer to the buffer of the two-stage model (see Supporting Figure S2).

Alternative one-stage models: Leaky drift-diffusion models

The dominance of the second stimulus suggests that the appropriate model must incorporate an information leak. We tested whether the data can be explained by two different versions leaky one-stage model. In the first model, the dynamics of the decision variable $X(t)$ is described by $dX = \nu dt + dW - X dt/\tau$, where ν is the drift rate, dW is a Wiener process and τ is the time constant of the leak. This drift-diffusion process is an Ornstein-Uhlenbeck process with decision bounds. The drift rate $\nu = c' \cdot stim(t)$ depends on the stimulus $stim(t)$ which is $+1$ during the presentation of stimulus 'A', -1 for stimulus 'B', and 0 otherwise. The magnitude of the drift rate is varied by setting c' to different values between 0.0 and 10 in steps of 2.5.

This model predicts increasing dominance of the first stimulus with increasing duration (Supporting Figure S3A). We found no parameter for which the second stimulus dominates. Thus, one-stage models with leaky evidence integration are not consistent with the experimental results.

As an alternative, we also tested a variant of the above model, in which the leak is removed at the offset of the stimulus, i.e. τ is finite during the stimulus and increased to infinity, $\tau \to \infty$, when it ends. For small noise and weak stimulus intensities, i.e. for small magnitudes c' of the drift, this is similar to using the output of a leaky stimulus integration as starting point for a non-leaky diffusion process with zero drift. Such a model generates a dominance of the second stimulus for intermediate stimulus durations: by the end of the stimulus, no decision bound is reached, but – on average – the diffusion variable is closer to the decision bound for the second stimulus, leading to bias in the decision (Supporting Figure S3B). For long stimuli, this dominance breaks down, because the decision bound can be reached already during the presentation of the first stimulus, leading to a dominance of the first stimulus.

Two-stage model

For long stimulus durations, the two-stage model smoothly converts into a one-stage model. To illustrate this, we studied the behavior of the model for long stimulus durations, where a one-stage model would predict a dominance of the first stimulus or, for the intermediate duration of 160 ms in our experiments, a weaker dominance of the second.

The model was fit to the data of experiment one. Fitting was done in two separate steps. First, we fit the leaky integrator of stage one and the drift-diffusion process of the extended two-stage model onto the reaction time distributions using the standard procedure to those conditions in which we found the model to work reasonably well (i.e. 20-80 ms total duration, but not 160 ms). Second, we keep the parameters found in the first step fixed and optimize T_{start} to fit the dominance across all conditions (i.e. 20-160 ms total duration). The fitting of T_{start} is done for each observer individually by minimizing the sum of the mean square error of the dominance across conditions (range of tested T_{start}: $0 \leq T_{start} - T_{\mathrm{pre}} \leq 120\,ms$) using 10.000 repetitions.

The model indeed shows a weaker dominance of the second stimulus for large stimulus durations (Supporting Figure S3C). It bears similarities to one-stage models using sensory pre-processing [1]. However, there are two marked differences: First, one-stage models using sensory pre-processing do not comprise a buffer that allows informed decision making to continue after the disappearance of the stimulus. Second, they start the drift-diffusion with stimulus onset (i.e. $T_{start} = T_{\mathrm{pre}}$). However, without these features the decision variable moves towards the decision bound for stimulus 'A' before dropping back to chance level. The decision variable does not continue towards the decision bound for stimulus 'B'. Therefore, these models also show a dominance of the first stimulus.

References

1. Purcell BA, Heitz RP, Cohen JY, Schall JD, Logan GD, et al. (2010) Neurally constrained modeling of perceptual decision making. Psychol Rev 117: 1113–1143.

Tables

Table S1. Evidence rates $W(S_T|I)$ in units of 1/ms.

stimulus	‘A’	‘B’	‘blank’
object *A*	4.08	3.92	-8.
object *B*	3.92	4.08	-8.
object *blank*	-8.	-8.	16.
